# A Novel Protein Subcellular Localization Method With CNN-XGBoost Model for Alzheimer's Disease

**DOI:** 10.3389/fgene.2018.00751

**Published:** 2019-01-18

**Authors:** Long Pang, Junjie Wang, Lingling Zhao, Chunyu Wang, Hui Zhan

**Affiliations:** ^1^Harbin Nebula Bioinformatics Technology Development Co., Ltd., Harbin, China; ^2^School of Computer Science and Technology, Harbin Institute of Technology, Harbin, China; ^3^School of Electronic Engineering, Heilongjiang University, Harbin, China

**Keywords:** protein subcellular localization, deep learning (DL), Conventional Neural Network (CNN), XGBoost, machine learning

## Abstract

The disorder distribution of protein in the compartment or organelle leads to many human diseases, including neurodegenerative diseases such as Alzheimer's disease. The prediction of protein subcellular localization play important roles in the understanding of the mechanism of protein function, pathogenes and disease therapy. This paper proposes a novel subcellular localization method by integrating the Convolutional Neural Network (CNN) and eXtreme Gradient Boosting (XGBoost), where CNN acts as a feature extractor to automatically obtain features from the original sequence information and a XGBoost classifier as a recognizer to identify the protein subcellular localization based on the output of the CNN. Experiments are implemented on three protein datasets. The results prove that the CNN-XGBoost method performs better than the general protein subcellular localization methods.

## 1. Introduction

The study of neurodegenerative diseases, specifically the Alzheimer's disease(AD) has gained great attention and been addressed widely (Cai et al., 2013; Hu et al., 2017a,b,c). The abnormalities and disorder distribution the compartment or organelle of tau protein and the beta-amyloid protein have been considered to contribute to the pathogenesis of AD. Protein subcellular localization prediction is an essential task in bioinformatics and plays import roles in the further understanding of the relationship among protein locations, their function exhibition, and nosogenesis (Liu et al., 2015; Cheng et al., 2016a, 2018a). Related predictive tools typically use the amino acid sequence information of the protein itself as input to output predicted protein cell sublocalization. It provides information on protein function and gene annotation to aid in the identification of drug targets. The two commonly used methods are: (1) homology-based method and (2) machine learning based method (Wu and Krishnan, 2011; Wu et al., 2014; Zeng et al., 2014; Cheng et al., 2017).

The homology-based method highly depends on the homology of protein sequences, and therefore performs worse for low protein sequence similarity (Wei et al., 2016; Cheng et al., 2018b). The machine learning based methods usually extract some features from the amino acid sequence of the protein (Cheng et al., 2016b; Hu et al., 2018), convert the sequence into a numerical vector, and then use a machine learning model to predict. For example, the most widely used WoLF PSORT software for eukaryotic proteins, characterized by the amino acid composition of the protein, gives the cellular sublocalization of the 32 proteins most similar to the input protein using the k-nearest neighbor algorithm (Horton et al., 2007). There also exist similar methods like BaCelLo (Pierleoni et al., 2006), YLoc (Briesemeister et al., 2010), iLoc-Hum (Chou et al., 2012), and Hum-mPLoc 3.0 (Zhou et al., 2016).

We believe that existing forecasting methods also have some room for improvement. First, the extracted sequence characteristics may not fully reflect the properties of the protein associated with the training task. Second, the current predictions only use information about the protein itself, without considering the interaction between proteins.

In recent years, deep learning has been proven to be a very powerful method by researchers in many fields (LeCun et al., 2015; Xu et al., 2017), like computer vision and natural language processing (Krizhevsky et al., 2012; Mikolov et al., 2013; Sutskever et al., 2014). CNN is an efficient deep learning method due to it can learn high-level features with neural networks. Recently, it also has attracted attentions from researchers and practitioners in bioinformatics. A prediction tool “DeepLoc” (Almagro Armenteros et al., 2017) based on deep learning was proposed with the end-to-end sequence-based model integrated recurrent neural networks (RNNs) with long short-term memory(LSTM) cells, attention models and convolutional neural networks(CNNs), and achieved a better accuracy compared with the traditional machine learning methods. However, the model structure is of high complication, sequentially has too many hyper-parameters to train. Moreover, the proteins in the dataset they constructed have been found to be highly homologous and therefore might provide an overly optimistic model evaluation (Gudenas, 2018). In addition, DeepLoc considers only one possible label for each protein, whereas the protein subcellular location belongs to a multi-label multi-class problem in general.

In this work, we propose a new framework for protein subcellular localization prediction by combining CNN and XGBoost. As an outstanding classifier and feature extractor, CNNs have achieved great success, especially in the field of image recognization. For the protein sequence, CNNs have ability to detect short motifs in the input sequence irrespectively of where they occur and automatically extract features from the original protein sequences. Inspired by this advantage, we also exploit CNN as the feature extractor but a new classifier XGBoost to replace the traditional classifiers connected like the soft-max classifier, since they can not well understand the extracted feature by CNN. XGBoost is an efficient implementation of gradient boosted decision trees (GBDT) due to its block structure to support the parallelization of tree construction. In GBDT, gradient boosting refers to a kind of ensemble technique creating new models to predict the residuals or errors of prior models and making the final decision by the summing up the predictions from all models. Meantime, gradient descent algorithm is also exploited to minimize the loss when adding new models.

The main contribution of our work includes the following aspects:

- We propose a new CNN-XGBoost model for prediction of the protein subcellular localization. The high-level features of protein sequence can be learned by a CNN that can be used by XGBoost classifier for prediction the localization of the subcellular of proteins.
- The experiments conducted on four real datasets consisting of protein sequences show that the proposed method achieves competitive performance.

## 2. Methods

**Figure 1:**
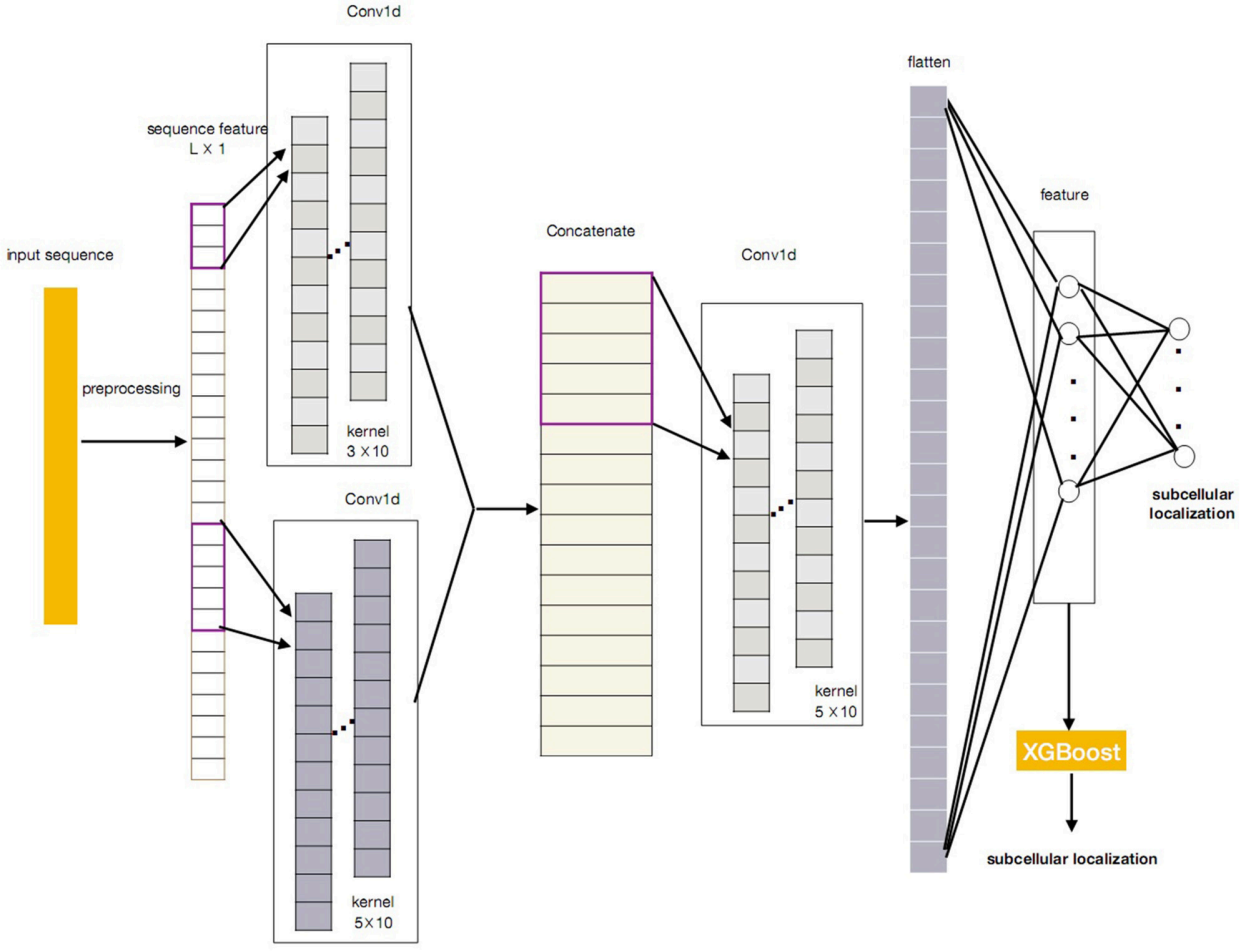
The framework of the CNN-XGBoost based protein subcellular location predictor.

In this paper, we propose a novel protein subcellular localization method by integrating the CNN and the XGBoost as a new model for possible application in the pathogenes verification of Alzheimer's disease. The general concept of CNN-XGBoost model is to add an XGboost after the feature layer of a CNN and replace the output layer of the CNN. Our CNN-XGBoost model can automatically extract featutue from the protein sequences and provides more precise localization results. Figure 1 illustrates the whole structure of the CNN-XGBoost model for protein subcellular localization.

### 2.1. Convolutional Neural Network

In the field of image analysis, the mask (or filter, or kernel) is an important construct. A *convolution* is an operation involving an initial image and the mask. The operation is equivalent to flipping the mask both vertically and horizontally and then visually placing it over each pixel in turn. The output is the sum over a pixel-wise product of the mask and the sub-image. Masks are usually symmetric, so flipping is unnecessary. Recall from signal processing, the *convolution* between two *f* and *g* is given by the following equation.

(1) $$\left(f*g\right)\left(t\right)≜\int_{-\infty }^{+\infty }f\left(\tau \right)g(t-\tau )d\tau$$

In image processing, a convolution between an image **I** and *kernel* **K** of size *d* × *d* centered at a given pixel (*x, y*) is defined as,

(2) $$\left(I*K\right)\left(x,y\right)=\sum_{i=1}^{d}\sum_{j=1}^{d}I(x+i-d/2,y+j-d/2)\times K(i,j)$$

Convolutional neural networks are a family of neural network architectures having at least one convolutional layer. *LeNet* is the original CNN network architecture bearing the name of Yann Lecun. Its architecture can be written as,

$$\begin{array}{rc} {\text{H}}_{1} & =\sigma \left(\text{X}*{\text{K}}^{\left(1\right)}\right)\;\text{(first convolutional layer)}\\ {\text{P}}_{1} & =\text{maxpool}\left({\text{H}}_{1}\right)\;\text{(first pooling layer)}\\ {\text{H}}_{2} & =\sigma \left({\text{P}}_{1}*{\text{K}}^{\left(2\right)}\right)\;\text{(second convolutional layer)}\\ {\text{P}}_{2} & =\text{maxpool}\left({\text{H}}_{2}\right)\;\text{(second pooling layer)}\\ {\text{F}}_{1} & =\sigma \left({\text{W}}^{\left(1\right)}{\text{P}}_{2}+{\text{b}}^{\left(1\right)}\right)\;\text{(first fully-connected layer)}\\ {\text{F}}_{2} & =\sigma \left({\text{W}}^{\left(2\right)}{\text{F}}_{1}+{\text{b}}^{\left(2\right)}\right)\;\text{(second fully-connected layer)}\\ \text{f}\left(\text{X}\right) & =\text{softmax}\left({\text{W}}^{\left(3\right)}{\text{F}}_{2}+{\text{b}}^{\left(3\right)}\right)\;\text{(output layer)} \end{array}$$

In this architecture, *convolutional layer* is the cornerstone of the CNN, which is a hidden layer where a square grid of weights is convolved with the input, just like an image mask. The output of the convolutional layer is akin to a convolved image. Next, the non-linear activation function, ReLu (REctified Linear Unit), is applied to zero-out any negative values. To reduce the dimension of the feature extracted from the convolutional layer, there is a *pooling* layer emulating *downsampling*. In general, each group of four values or pixels is replaced by the maximum (sometimes the mean) of the four, leaving a single most intense pixel. This pooling method is known as *max pooling*. This sequence of CONV->RELU->POOLlayers may be repeated multiple times to create a deep architecture. Finally, a few fully-connected layers round off the architecture. Though it seems far more sophisticated than a MLP, it can be shown that a CNN can be represented as a classical fully-connected neural network. For example, a convolutional layer can be represented as a sparse fully-connected layer. Various techniques have been developed for training these vast models, for example momentum optimizers, weight initialization, batch normalization, and dropout.

Convolutional Neural Networks are the current state-of-the-art in many computer vision tasks. In addition to image classification, their great success has attracted wide attention in many fields. It has been found that using a pre-trained CNN as a general-purpose feature extractor for a simple linear model can yield significant improvements over even the most meticulously hand-crafted feature engineering.

The protein subcellular localization problem can be viewed as a multi-label multi-class classification task. Unlike the ordinary deep learning methods for multi-classification problems, in our method, we need to change the loss function. The most intuitive way is to extend the cross-entropy loss. The cross-entropy loss function is defined by

(3) $$\underset{\Theta }{\min}-\frac{1}{n}\sum_{i=1}^{n}\sum_{j=1}^{L}y_{i,j}\text{log}({\hat{p}}_{ij})=-\frac{1}{n}\sum_{i=1}^{n}\sum_{j\in y_{i}^{+}}\frac{1}{\left|y_{i}^{+}\right|}log({\hat{p}}_{ij})$$

where Θ denotes the parameters of CNN model, $y_{i}^{+}$ is a set that contains the relevant localization of protein *i* and ${\hat{p}}_{ij}$ is the result for protein *i* on localization *j*, through a softmax activation:

(4) $${\hat{p}}_{ij}=\frac{\exp\left(f_{j}\left(x_{i}\right)\right)}{\sum_{j'}^{L}\exp\left(f_{j'}\left(x_{i}\right)\right)}$$

Instead of using the cross-entropy loss function, the binary cross-entropy loss (BCE) over sigmoid activation has shown better performance when applied into multi-label task. The binary cross-entropy loss is

(5) $$\underset{\Theta }{\min}-\frac{1}{n}\overset{n}{\underset{i=1}{\sum }}\sum_{j=1}^{L}\left[y_{i,j}\log\left(\sigma \left(f_{ij}\right)\right)+\left(1-y_{ij}\right)\log\left(1-\sigma \left(f_{ij}\right)\right)\right]$$

where $\sigma \left(x\right)=\frac{1}{1+e^{-x}}$

### 2.2. Tree Boosting and XGBoost

Tree boosting is a learning method to enhance the classification ability of weak classifiers by iteratively adding new decision trees to the ensembles of decision trees. Let $D=\left\{\left(x_{i},y_{i}\right)\right\}\left(|D|=n,x_{i}\in {\mathbb{R}}^{m},y_{i}\in {\mathbb{R}}^{n}\right)$ denotes a dataset with *n* classes and *m* feature. Then the prediction of a tree boosting for a (*x*_*i*_, *y*_*i*_) is given by

(6) $$\begin{array}{l} {\hat{y}}_{i}=g_{A}(x_{i})=\sum_{j=1}^{M}g_{j}(x_{i}) \end{array}$$

where *g*_*j*_(*x*_*i*_) = *w*_*q*_(*x*_*i*_) is the prediction of the *j*-th decision tree with leaf weights *w*_*q*_ on a datapoint *x*_*i*_, and *M* is the number of members in the ensemble.

It is well-known that the decision tree tends to overfit when the decision tree is fully grown. Thus, the set prediction function of decision trees *g*_*j*_ can be learned by minimizing the objective function

(7) $$C(x,g_{A})=\sum_{i=1}^{N}l(y_{i},{\hat{y}}_{i})+\sum_{j=1}^{M}\Omega (g_{j})$$

where *l*_*i*_(*y*_*i*_, ŷ_*i*_) is a term which measures the goodness of the prediction ŷ_*i*_ and the object *y*_*i*_. Ω(*g*_*j*_) is a regularization term that does not depend on the data.

XGBoost implements a parallel tree boosting in a fast and accurate way. In XGBoost, the regularization function is chosen to be

(8) $$\begin{array}{l} \Omega (g)=\gamma T+\frac{\lambda }{2}\sum_{l=1}^{T}w_{l}^{2} \end{array}$$

with γ and λ regularization parameters that must be chosen appropriately. Notice this regularization penalizes both large weights on the leaves (similar to *L*^2^-regularization) and has large partitions.

As mentioned above, the tree boosting iteratively enlarges the ensemble of decision trees, then the prediction of the *t*-th iteration can be defined as

(9) $${\hat{y}}_{i}^{\left(t\right)}=\sum_{j=1}^{t}g_{j}\left(x_{i}\right)={\hat{y}}_{i}^{\left(t-1\right)}+g_{t}\left(x_{i}\right)$$

The objective function (7) at step *t* can be modified as

(10) $$\begin{array}{ll} C_{t}= & \sum_{i=1}^{N}l\left(y_{i},{\hat{y}}_{i}^{\left(t-1\right)}+g_{t}\left(x_{t}\right)\right)+\Omega \left(g_{t}\right) \end{array}$$

Apply a Taylor expansion on the objective function (10) to second order and then the final objective function at step *t* can be approximated as

(11) $$C_{t}\approx C_{t-1}+\Delta C_{t}$$

(12) $$\;=\;C_{t-1}+b_{i}l\left(y_{i},{\hat{y}}_{i}^{\left(t-1\right)}\right)g_{t}\left(x_{i}\right)+\frac{1}{2}a_{i}g_{t}{\left(x_{i}\right)}^{2}+\Omega \left(g_{t}\right)$$

where

(13) $$a_{i}=\partial_{{\hat{y}}_{i}^{\left(t-1\right)}}l\left(y_{i},{\hat{y}}_{i}^{\left(t-1\right)}\right)$$

(14) $$b_{i}=\partial_{{\hat{y}}_{i}^{\left(t-1\right)}}^{2}l\left(y_{i},{\hat{y}}_{i}^{\left(t-1\right)}\right)$$

Let *j*:*I*_*j*_ = {*i*:*q*_*t*_(*x*_*i*_) = *j*} denotes the set of point *x*_*i*_ mapped to leaf, $B_{j}=\underset{i\in I_{j}}{\sum }b_{i}$ and $A_{j}=\underset{i\in I_{j}}{\sum }a_{i}$. Then we can rewrite the $\Delta C_{t}$ as

(15) $$\begin{array}{r} \Delta C_{t}=\overset{T}{\underset{j=1}{\sum }}[B_{j}w_{j}+\frac{1}{2}\left(A_{j}+\lambda_{j}\right)w_{j}^{2}]+\lambda T \end{array}$$

To find the optimal weight *w*_*j*_ of leaf *j* for a fixed tree structure, *q*(*x*) can be obtained by applying the following equation

(16) $$w_{j}^{opt}=-\frac{B_{j}}{A_{j}+\lambda }$$

plugging back into $\Delta C_{t}$ gives

(17) $$\Delta C_{t}^{opt}=-\frac{1}{2}\sum_{j=1}^{K}\frac{B_{j}^{2}}{A_{j}+\lambda }+\gamma T$$

It is clear that $\Delta C_{t}^{opt}$ measures the in-sample performance of *g*_*t*_ and we should find the decision tree that minimizes this value. However, in practice, this is impossible to enumerate all possible trees over the data and find the tree which can minimize $\Delta C_{t}^{opt}$. Instead, an approximate greedy algorithm runs to optimize one level of the tree at a time by trying to find optimal splits of the data, leading to a tree with a local minimum of $\Delta C_{t}^{opt}$, which is then added to the ensemble.

For the multi-label multi-class classification problem, we utilize XGBoost as classifiers and adopt the binary relevance strategy (Boutell et al., 2004) to construct *m* binary classifiers.

### 2.3. CNN-XGBoost Model

Figure 1 gives the overall structure of the CNN-XGBoost model for protein subcellular location prediction. The input of the model is a one-dimensional vector and constructed by the position specific scoring matrices (PSSM) and proteins interaction scoring matrix which are extracted from STRING and GO terms semantic similarities. On this basis, a protein can be expressed as *L* × 1 vector (*L* is the number of sequences in training set), analog image data equivalent to a protein is a one-dimensional “image” with 1 channels. So the input is a *L* × 1 matrix.

After obtaining the proper feature representations by the trained CNN, compared with the classic CNN, our CNN-XGBoost model replaces the soft-max layer of CNN with XGBoost to predict the localization of subcellular of proteins, which enables features automatically obtained from input and provides more precise and efficient classification.

## 3. Results

### 3.1. Dataset

To verify the performance of our method, we employ three protein datasets: the Hum-mPloc3.0, the BaCelLo animals, and the Hoglund. Table 1 gives the details of these datasets. The train set of Hum-mPloc 3.0 consists of 3,122 proteins and 1,023 proteins own more than one label. The test set of Hum-mPloc 3.0 consists of 379 proteins, among which 120 proteins belong to multi-label proteins. Each protein in Hum-mPloc 3.0 is assigned at least one label of 12 subcellular locations (Centrosome, Cytoplasm, Cytoskeleton, Endoplasmic reticulum, Endosome, Extracellular, Golgi apparatus, Lysosome, Mitochondrion, Nucleus, Peroxisome, and Plasma membrane).

**Table 1:** Dataset Summary.

	**Hum-mLoc 3.0**		**BaCelLo**		**Hoglund**	
	**Training**	**Testing**	**Training**	**Testing**	**Training**	**Testing**
No. Proteins	3,126	379	2,597	576	5,959	158
No. Labels	4,229	541	2,597	576	5,959	158
No.Locations	12		4		6	

For the BaCelLo dataset, there are four subcellular locations: Cytoplasm, Mitochondrion, Nucleus, and Secreted. The size of the training set is set to 2,597 and the testing set consists of 576 proteins. All the proteins of BaCelLo dataset are of a single label. In the Hoglund dataset, the training set includes nine subcellular locations (Nucleus, Cytoplasm, Mitochondrion, Endoplasmic reticulum, Golgi apparatus, Peroxisome, Plasma membrane, Extracellular space, Lysosome, and Vacuole), and the test consists of 158 proteins with six subcellular locations (Endoplasmic reticulum, Golgi apparatus, Peroxisome, Plasma membrane, Extracellular space, and Lysosome).

### 3.2. Measurements

A widely-applied method for evaluating a mutli-label multi-class classifier is to compute the ACC and F1 values. ACC is the average of *ACC*_*x*_*i*__ of all proteins in the testing set, calculated for protein *x*_*i*_ is

(18) $${\text{ACC}}_{x_{i}}=\frac{{\text{TP}}_{x_{i}}}{{\text{TP}}_{x_{i}}+{\text{FP}}_{x_{i}}+{\text{FN}}_{x_{i}}}$$

where TP, FP, and FN are true positive, false positive, and false negative, respectively. The F1 score considers both the harmonic mean of precision and recall of subcellular location *y*_*j*_, defined as follows:

(19) $$\begin{array}{r} \begin{array}{cc} {\text{precision}}_{y_{j}} & =\frac{\sum_{x_{i}\in P_{j}}\frac{{\text{TP}}_{x_{i}}}{{\text{TP}}_{x_{i}}+{\text{FP}}_{x_{i}}}}{\left|P_{j}\right|}\\ {\text{recall}}_{y_{j}} & =\frac{\sum_{x_{i}\in T_{j}}\frac{{\text{TP}}_{x_{i}}}{{\text{TP}}_{x_{i}}+{\text{FN}}_{x_{i}}}}{\left|T_{j}\right|}\\ {\text{F1}}_{y_{j}} & =\frac{2\times {\text{precision}}_{y_{j}}\times {\text{recall}}_{y_{j}}}{{\text{recall}}_{y_{j}}+{\text{precision}}_{y_{j}}} \end{array} \end{array}$$

where *T*_*j*_ and *P*_*j*_ are the set of proteins for true location *y*_*j*_ and the set of proteins for predicted locations *y*_*j*_ respectively.

### 3.3. Results and Discussions

**Figure 2:**
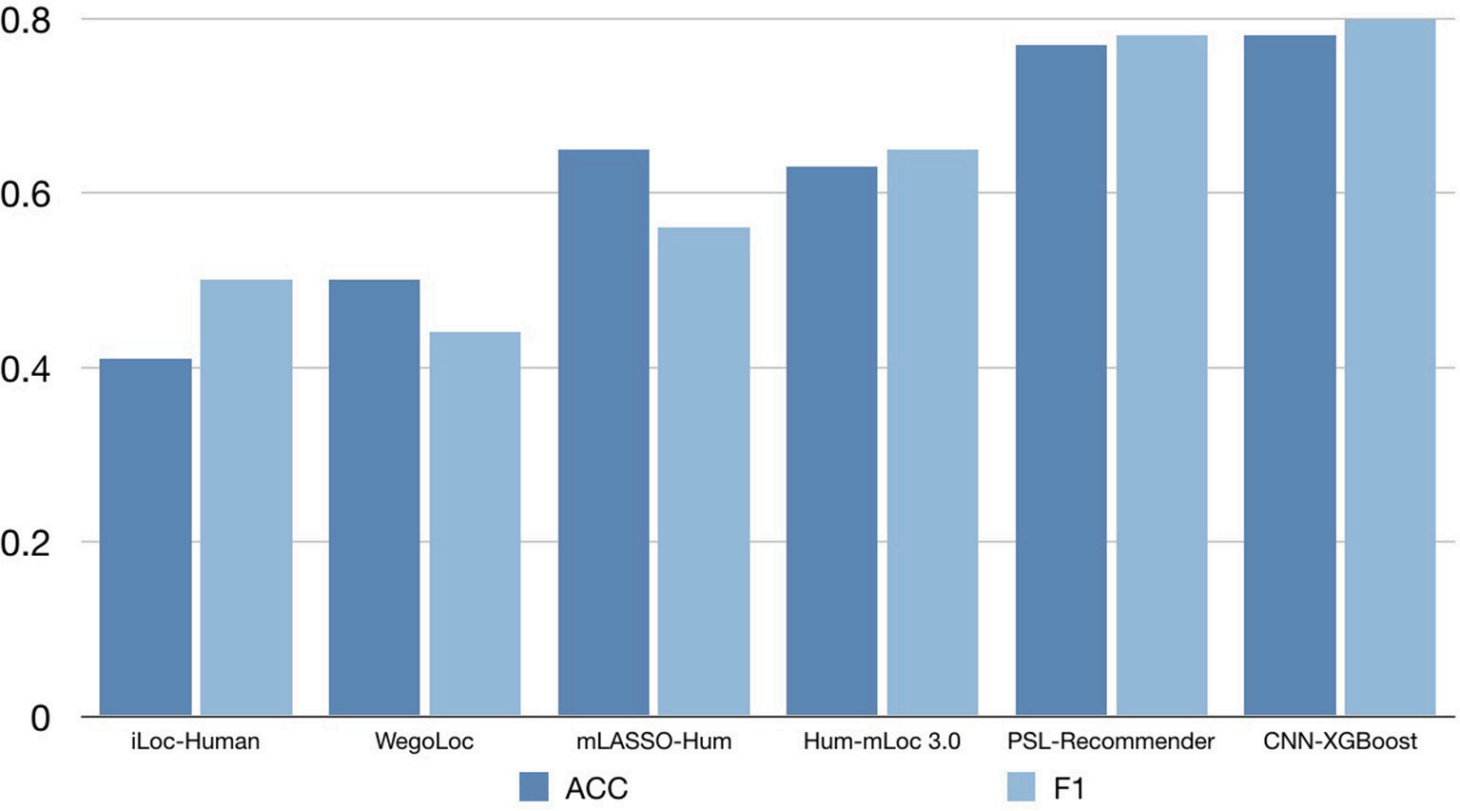
The accuracy comparison on the Hum-mPloc 3.0 data set.

To verify the performance of our approach, some typical protein subcellular location tools including Hum-mPLoc 3.0 (Zhou et al., 2016), YLoc+ (Briesemeister et al., 2010), iLoc-Hum (Chou et al., 2012), WegoLoc (Chi and Nam, 2012), mLASSO-Hum (Wan et al., 2015), and PSL-Recommender (Jamali et al., 2018) were compared to our method. The F1 score and ACC for each subcellular localization are summarized in Table 2 and Figure 2 for Hum-mploc 3.0 dataset. As seen in Table 2 and Figure 2, the CNN-XGBoost outperforms the mean value of F1 score and ACC of all other methods. Also, in 7 out of 12 subcellular locations, CNN-XGBoost has the best performance among all the methods while in the other three locations it has the second best performance. It is only in centrosome and endosome that CNN-XGBoost shows unsatisfactory results. As seen in Table 3, the CNN-XGBoost slightly outperforms the second best method by both mean F1 score and ACC.

**Table 2:** Comparision of CNN-XGBoost on Hum-mPloc 3.0 dataset with other methods.

**Location**	**iLoc-Human**			**WegoLoc**			**mLASSO-Hum**			**Hum-mLoc 3.0**			**PSL-Recommender**			**CNN-XGBoost**		
	**pre**	**re**	**F1**	**pre**	**re**	**F1**	**pre**	**re**	**F1**	**pre**	**re**	**F1**	**pre**	**re**	**F1**	**pre**	**re**	**F1**
Centrosome	0	0	0	0.75	0.14	0.23	0.59	0.59	0.59	0.75	0.55	0.63	0.94	0.75	**0.83**	0.79	0.50	0.61
Cytoplasm	0.5	0.54	0.52	0.69	0.53	0.60	0.93	0.51	0.66	0.76	0.73	0.74	0.79	0.81	0.80	0.85	0.89	**0.87**
Cytoskeleton	0	0	0	0.32	0.34	0.33	0.9	0.22	0.35	0.8	0.68	0.74	0.93	0.77	0.84	0.89	0.80	**0.85**
ER	0	0	0	0.73	0.2	0.31	0.74	0.49	0.59	0.83	0.37	0.51	0.9	0.7	0.79	0.97	0.71	**0.82**
Endosome	0	0	0	0.25	0.07	0.11	0.38	0.2	0.26	0.58	0.47	**0.52**	0.57	0.37	0.45	0.80	0.27	0.40
Extracellular	0.62	0.62	0.62	0.67	0.77	**0.71**	0.16	0.69	0.26	0.5	0.46	0.48	0.66	0.71	0.68	0.80	0.62	0.70
Golgi apparatus	0.6	0.3	0.4	0.6	0.15	0.24	0.72	0.65	0.68	0.69	0.45	0.55	0.88	0.61	**0.72**	0.80	0.60	0.69
Lysosome	0.5	0.13	0.2	0.2	0.13	0.15	0.55	0.75	0.63	0.71	0.63	0.67	1	0.55	0.71	1.00	0.75	**0.86**
Mitochondrion	0.95	0.33	0.49	0.79	0.73	0.76	0.83	0.88	0.85	0.78	0.75	0.76	0.92	0.88	**0.90**	0.96	0.80	0.87
Nucleus	0.54	0.7	0.61	0.65	0.64	0.64	0.85	0.7	0.76	0.75	0.71	0.73	0.81	0.92	**0.87**	0.83	0.91	**0.87**
Peroxisome	1	0.5	0.67	0.5	1	0.67	0.29	1	0.44	1	1	**1**	1	1	**1**	1	1	**1**
Plasma membrane	0.42	0.33	0.37	0.44	0.53	0.48	0.58	0.56	0.57	0.65	0.44	0.52	0.78	0.74	0.76	0.89	0.75	**0.81**
ACC-mean	0.41			0.50			0.65			0.63			0.77			**0.78**		
F1-mean	0.32			0.44			0.56			0.65			0.78			**0.80**		

*The bold marks the first best result and the underline marks the second best result*.

**Table 3:** Comparison of CNN-XGBoost ACC/F1-mean on other proteins datasets with other methods.

	**BaCelLo**	**Hoglund**
MultiLoc2-LowRes	0.73/0.76	–
MultiLoc2-HighRes	0.68/0.71	0.57/0.41
BaCelLo	0.64/0.66	–
Hum-mPloc 3.0	0.86/0.84	0.64/0.59
PSL-Recommender	0.94/0.92	0.92/0.90
CNN-XGBoost	**0.94/0.94**	**0.94/0.92**

*The bold marks the first best result and the underline marks the second best result*.

In addition, we also evaluated our method on the DeepLoc dataset, compared to the DeepLoc, our method provides slightly better prediction with significantly lighter model, meanwhile, it is known that DeepLoc can not handle multilabel multiclass problem, whereas our method still shows outstanding performance.

## 4. Conclusions

In order to make balance of the classification performance and the complexity when training the model for the protein subcellular location in Alzheimer's disease, this paper proposes a prediction framework integrating CNN and XGBoost, taking advantage of the outstanding ability of feature expression of CNN, and the good classification performance of XGBoost. Experiments are implemented on the Hum-mPloc3.0, the BaCelLo animals, and the Hoglund database, and the results demonstrate that the new method outperforms the typical machine learning based tools. Further work will focus on the verification of our model on more datasets, especially the datasets related to Alzheimer's disease, and the optimization of the structure of CNN utilized in the model.

## Author Contributions

All authors listed have made a substantial, direct and intellectual contribution to the work, and approved it for publication.

### Conflict of Interest Statement

The authors declare that the research was conducted in the absence of any commercial or financial relationships that could be construed as a potential conflict of interest.
